# Ionic Liquid‐Mediated Transdermal Delivery of Thrombosis‐Detecting Nanosensors

**DOI:** 10.1002/adhm.202102685

**Published:** 2022-02-27

**Authors:** Ahmet Bekdemir, Eden E. L. Tanner, Jesse Kirkpatrick, Ava P. Soleimany, Samir Mitragotri, Sangeeta N. Bhatia

**Affiliations:** ^1^ Harvard–MIT Division of Health Sciences and Technology Institute for Medical Engineering and Science Massachusetts Institute of Technology Cambridge MA 02139 USA; ^2^ School of Engineering and Applied Sciences Harvard University Cambridge MA 02138 USA; ^3^ Now at Department of Chemistry and Biochemistry University of Mississippi Oxford MS 38677 USA; ^4^ Koch Institute for Integrative Cancer Research Massachusetts Institute of Technology Cambridge MA 02139 USA; ^5^ Harvard Graduate Program in Biophysics Harvard University Cambridge MA 02138 USA; ^6^ Wyss Institute of Biologically Inspired Engineering Harvard University Cambridge MA 02138 USA; ^7^ Department of Electrical Engineering and Computer Science Massachusetts Institute of Technology Cambridge MA 02139 USA; ^8^ Broad Institute of Massachusetts Institute of Technology and Harvard Cambridge MA 02139 USA; ^9^ Department of Medicine, Brigham and Women's Hospital Harvard Medical School Boston MA 02115 USA; ^10^ Wyss Institute Harvard University Boston MA 02115 USA; ^11^ Howard Hughes Medical Institute Cambridge MA 02138 USA

**Keywords:** activity‐based nanosensors, drug delivery, ionic liquids, nanoparticles, protease, thrombosis, transdermal delivery

## Abstract

Blood clotting disorders such as pulmonary embolism are associated with high morbidity and mortality. A large portion of thrombotic events occur postoperative and after hospital discharge. Therefore, easily applicable, noninvasive, and long‐term monitoring of thrombosis occurrence is critical for urgent clinical intervention. Here, the use is proposed of ionic liquids as a skin transport facilitator to deliver thrombin‐sensitive nanosensors that enable prolonged monitoring of pulmonary embolism. Co‐formulation of nanosensors with choline and geranic acid (CAGE) ionic liquids demonstrates significant transdermal diffusion into the dermis of the skin and provides sustained release into the blood throughout 72 h. Upon reaching the systemic circulation, the nanosensors release reporter molecules into the urine by responding to activation of the clotting cascade and retain a diagnostic power for 24 h in an acute pulmonary embolism mouse model. These results demonstrate a proof‐of‐concept disease monitoring system that can be topically applied by patients and potentially reduce mortality and high cost of hospitalization.

## Graphical abstract

Topical delivery of diagnostic nanosensors into the circulation have been achieved with ionic liquids. This nanosensor‐ionic liquid formulation is shown to detect acute pulmonary thromboembolism in a sustainable fashion which enables continuous monitoring. This is a proof‐of‐concept application of transdermal delivery of diagnostic nanomaterials for disease detection in a non‐invasive method.

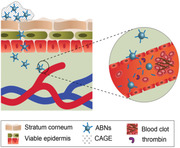

## 1 Introduction

Thrombosis and thromboembolism are life‐threatening medical complications that present high morbidity and mortality, particularly for postoperative patients.^[^ ^1^ ^]^ The continual monitoring for thrombosis occurrence through surveying prothrombin fragment levels in the blood is a common practice in hospital settings.^[^ ^2^ ^]^ After hospital discharge, however, monitoring for thrombosis is limited to subjective assessment of symptoms, such as shortness of breath and chest pain, which are nonquantitative and nonspecific.^[^ ^3^ ^]^ Since the risk of embolism is highly time‐dependent,^[^ ^4^ ^]^ constant and non‐invasive monitoring of thrombosis is critical for urgent and timely intervention. Recent studies that leverage dysregulated protease activity during thrombosis demonstrated potential diagnostic nanosensor system in thromboplastin injected mouse models. Thrombin‐sensitive peptides conjugated nanoparticles have been shown to generate urinary reporters that can discriminate pulmonary embolism from healthy conditions.^[^ ^5^, ^6^ ^]^ Despite the promise of these thrombosis detecting nanosensors (TDNs), current administration methods are limited to intravenous or subcutaneous injection which usually require healthcare professionals. In addition, nanosensors should only be injected shortly before or during the occurrence of thrombosis which is not a feasible clinical scenario. A transdermal delivery approach, on the other hand, would expand the utility of nanosensors beyond infusion centers and enable testing in remote locations. Furthermore, sustained release of such synthetic biomarkers could enable longitudinal monitoring for medical emergencies in high‐risk populations, such as thromboembolism in hypercoagulable patients after surgery or prolonged hospitalizations.^[^ ^7^ ^]^ In this work, we sought to assess whether ionic liquids could enable topical application of TDNs and impart serial monitoring for pulmonary emboli in a thromboplastin injected mouse model.

Macromolecule transport through the skin barrier presents a significant challenge.^[^ ^8^ ^]^ While device‐based strategies such as laser ablation, sonophoresis, iontophoresis, and microneedles have offered attractive solutions to enhance transdermal transport of large molecules, intrinsic hurdles regarding toxicity, and the application area on the body limit their use in a broader context.^[^ ^9^ ^]^ Recently, ionic liquids have been proposed as promising alternatives to enable diffusion of protein‐based therapeutics through the skin, which have exhibited efficient biological responses in the blood.^[^ ^10^, ^11^ ^]^ However, ionic liquids have not been shown to enhance transdermal transport of diagnostic agents, or of any nanomaterials, for that matter. Here, we demonstrate the use of ionic liquids to deliver diagnostic nanomaterials, and use enzyme‐responsive TDNs as a proof‐of‐principle application for prolonged thrombosis detection.^[^ ^12^ ^]^ To achieve this goal, we leveraged a choline and geranic acid (CAGE)‐based ionic liquid/deep eutectic solvent to solubilize and transdermally deliver nanosensors that can detect thrombosis in a mouse model of acute pulmonary embolism. CAGE, when synthesized at a 1:2 molar ratio of choline and geranic acid, respectively, has previously demonstrated superior insulin transport capability and achieved therapeutically relevant blood glucose levels without apparent toxicity.^[^ ^13^ ^]^ Considering that our polyethylene glycol (PEG) nanosensors have a molecular weight comparable to hexameric insulin, they may also be a candidate for topical delivery with the same CAGE formulation. These nanosensors carry thrombin‐cleavable peptide substrates appended to renal‐clearable reporter molecules. Upon thrombin‐mediated substrate cleavage at the site of disease, liberated reporter fragments are filtered through the kidneys and concentrated in urine, which can then be analyzed via fluorescence for detection of disease.

## 2 Results and Discussion

**Figure 1:**
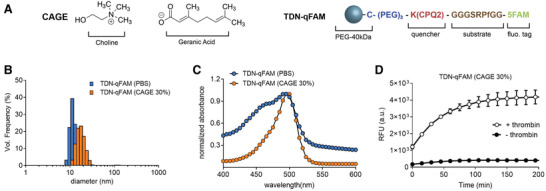
A) Chemical components of CAGE ionic liquid and schematic representation of TDN‐qFAM nanoparticles with thrombin substrate sequence. B) Dynamic light scattering and C) absorbance spectra of TDN‐qFAM in PBS and CAGE solutions. c) Fluorescence dequenching experiment for enzyme cleavage kinetics in the presence (empty circles) and absence (full circles) of recombinant thrombin in the TDN‐qFAM/CAGE solution. Error bars represent standard deviation of triplicate measurements. Invisible error bars are due to standard deviations that are lower than the marker size.

We first show that our nanosensors retain their stability and functionality in a complex ionic liquid environment.^[^ ^14^ ^]^ To assess the solubility, stability, and activity of our nanosensors in CAGE, we conjugated a thrombin substrate (–GGfPRSGGG–) to 40 kDa PEG nanoparticles at an 8:1 substrate:nanoparticle ratio. The substrates were flanked by 5‐carboxyfluorescein (5‐FAM) and a Förster resonance energy transfer quencher molecule (CPQ2) in close proximity, such that proteolytic cleavage would result in fluorescence signal (nanoparticle was denoted as TDN‐qFAM, **Figure** **1**A). Dynamic light scattering size analysis did not exhibit any significant aggregation associated size increase (>100 nm) that indicates the stability of TNDs in 30% CAGE solution (Figure 1B). A slight increase of the mean hydrodynamic size of TDN‐qFAM when dissolved in CAGE likely originated from additional CAGE molecules associating with the nanoparticle surface. In addition, the colloidal stability of TDN‐qFAM was assessed by the absorption spectrum of TDN‐qFAM in the presence and absence of CAGE after 24 h of room temperature storage (Figure 1C). The absorption maximum at 495 nm that corresponds to 5‐FAM absorption was virtually unchanged after storage. We then investigated whether the presence of CAGE components influenced the stability of the peptide substrate and the activity of thrombin against TDN‐qFAM. Fluorescence measurements upon addition of recombinant thrombin into the TDN‐qFAM solution in 30% CAGE showed a clear cleavage profile with gradual increase in fluorescence intensity (Figure 1D). This cleavage appeared to be dependent on the presence of thrombin, since no cleavage was observed in the absence of recombinant enzyme. This finding also indicated that CAGE alone did not compromise the stability of the peptides and results in nonspecific cleavage. Importantly, when TDN‐qFAM was incubated in a mouse serum sample, endogenous thrombin activity was partially hindered by the presence of 30% CAGE (Figure S1, Supporting Information). However, upon stepwise dilution of CAGE with phosphate buffered saline (PBS), the functionality of TDN‐qFAM against thrombin cleavage was improved. These results suggest that nanosensors can be coformulated with CAGE in a sufficiently stable fashion without inhibiting the proteolytic cleavage of thrombin‐sensitive peptide substrates.

**Figure 2:**
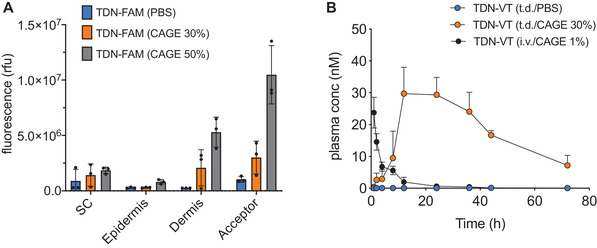
A) Quantification of TDN‐FAM extracted from different layers of mouse skin via fluorescence readout. SC: Stratum Corneum. Bars represent the mean value; error bars represent standard deviations, and circles represent individual results of 3 different skin samples. B) In vivo pharmacokinetics profile of topically administered TDN‐VT nanoparticles with PBS (blue circles), CAGE (orange circles), and intravenously injected with 1% CAGE (black circles); *n* = 6 mice per group; mean±s.d.

Next, we validated the skin penetration of the nanosensors by coformulating unquenched, 5‐FAM labelled, thrombin substrate‐conjugated PEG nanoparticles (TDN‐FAM, Table S1, Supporting Information) and 30% CAGE solution. Ex vivo conditions were established by incubating TDN‐FAM/CAGE with mouse skin samples that were stabilized in a Franz diffusion cell set‐up.^[^ ^14^, ^15^ ^]^ Three main layers of skin samples, namely stratum corneum, viable epidermis, and dermis, as well as the PBS solution underneath the skin (acceptor solution), were collected, and nanosensor concentration was quantified via fluorescence spectroscopy. The stratum corneum and viable epidermis layers captured similar amounts of TDN‐FAM nanoparticles when administered in either PBS or 30% CAGE solution (**Figure** **2**A). However, the dermis layer and acceptor solution exhibited markedly increased signal from TDN‐FAM when administered with 30% CAGE. Increasing the CAGE content to 50% resulted even higher recovery of nanosensors from dermis and acceptor layers This indicates that ionic liquids promote the transport of nanosensors into the deep skin of an ex vivo mouse skin model in a concentration dependent way.

**Figure 3:**
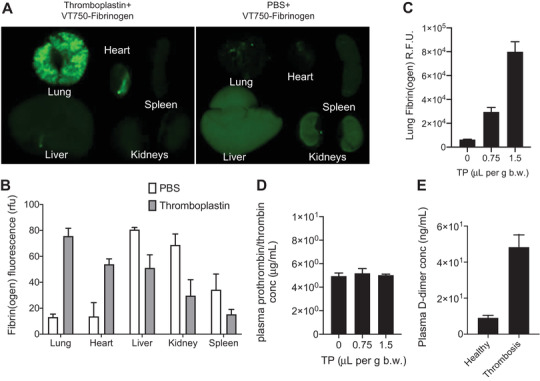
A) Fluorescence imaging of organs of mice collected after VT750‐Fibrinogen injection, either together with thromboplastin (0.75 µL per g body weight, left) or with PBS as a control (right). B) Biodistribution of fibrin(ogen) in Swiss Webster mice via VT750‐Fibrinogen with or without thromboplastin (*n* = 3 mice per group, mean ±s.d.). C) The dose response of thromboplastin measured by lung deposited fibrinogen, quantified as the fluorescent intensity of the images of collected organs (*n* = 3 mice per group, mean ±s.d.). D) Plasma concentrations of total prothrombin/thrombin levels with different doses of thromboplastin measured by ELISA (*n* = 3 mice per group, mean ±s.d.). E) Plasma levels of D‐dimer degradation product of fibrin in healthy (PBS control) versus thrombotic mice (thromboplastin injected, 0.75 µL per g body weight) measured by ELISA (*n* = 3 mice per group, mean ±s.d.).

The dermis is composed of dense, irregular connective tissue that accommodates a diverse array of epithelial and immune cells as well as sweat glands, hair follicles, and blood vessels. Although our ex vivo diffusion results demonstrated the transport of nanosensors through the layers of mouse skin, it did not provide insight as to whether the in vivo skin microenvironment would allow the nanosensors to partition into dermal blood vessels, thereby enabling systemic circulation. To assess whether the nanosensors could diffuse into the vascular system, we conjugated 40 kDa PEG nanoparticles with near‐infrared VivoTag750 dye (TDN‐VT), solvated them in a 30% CAGE solution, and topically administered onto a 1 cm^2^ dorsal skin area of Swiss Webster mice. Subsequent blood collection and plasma fluorescence analysis revealed the presence of TDN‐VT nanoparticles in the blood as early as 1 h after administration, and peaking at around 12 h (Figure 2B). Importantly, TDN‐VT nanoparticles were still present in the blood even after 72 h, indicating a prolonged and sustained delivery of nanoparticles into the circulation from the skin reservoir. In contrast, TDN‐VT nanoparticles administered in PBS exhibited negligible fluorescence signal in the blood at all tested time points (**Figure** **3**A; Figure S2, Supporting Information). When intravenously administered, nanoparticles were quickly cleared from circulation within a few hours, presenting vastly different pharmacokinetic profile than transdermal delivery.

Having established that thrombin‐sensitive nanosensors were able to efficiently reach systemic circulation after transdermal delivery with ionic liquids, we further inquired whether they could be used to actively monitor for thromboemboli in an acute thrombosis mouse model. To this end, we leveraged a mouse model of pulmonary embolism, in which intravenous injection of thromboplastin results in activation of coagulation via the extrinsic pathway and formation of clots that embolize to the lungs.^[^ ^16^ ^]^ To characterize this model, we coadministered fluorescently‐labelled fibrinogen and thromboplastin and collected the lung, liver, kidney, and spleen after 30 min. We found that lung fluorescence increased in response to thromboplastin administration, verifying that thromboplastin induced the formation of fibrin clots that embolized to the lung (Figure 3A). While biodistribution of fluorescent fibrinogen in healthy mice predominantly concentrated in the liver and the kidneys, thromboplastin resulted embolism in the lung and the heart (Figure 3B). In addition, the amount of fibrin clots found in the lungs were thromboplastin dose‐dependent and created higher clot burden with high dose of thromboplastin (Figure 3C). The plasma levels of total prothrombin/thrombin did not change upon induction of thrombosis that also corroborates the mechanistic basis of blood clotting due to conversion of prothrombin to thrombin instead of upregulation of the thrombin or prothrombin (Figure 3D). Additional ELISA test for D‐dimer, a degradation product of fibrin by plasmin, validated the elevated levels of D‐dimer in thrombosis induced mice and confirmed the diseased state (Figure 3E).

**Figure 4:**
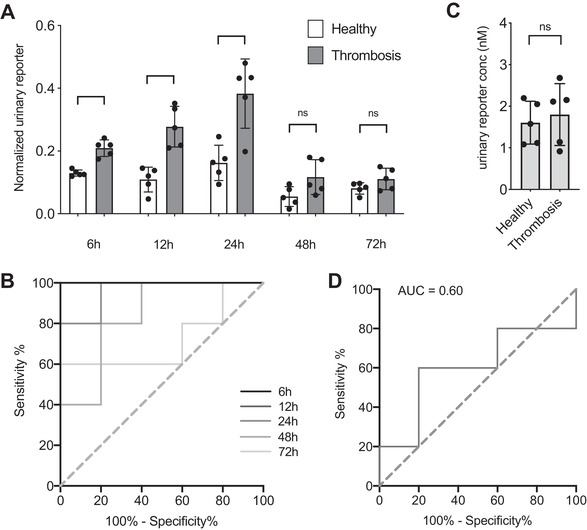
A) Urinary reporter concentrations relative to plasma TDN‐Cy7 concentrations for thromboplastin‐injected (Thrombosis) and healthy control mice at different time points after nanosensor administration (mean ± s.d., *n* = 5 mice per group; *P < 0.05 and **P < 0.01, two‐tailed Mann–Whitney's *t*‐test). B) Receiver operating characteristic (ROC) curves calculated from data at panel (A), for 6 h (AUC = 1.00), 12 h (AUC = 1.00), 24 h (AUC = 0.96), 48 h (AUC = 0.84), 72 h (AUC = 0.72). C) Urinary reporter concentrations in healthy versus thrombosis mice 6 h after intravenous injection of TDN‐Cy7 (mean ± s.d., *n* = 5 mice per group; ns: not significant). D) ROC curve calculated from data at panel (C) (AUC = 0.60).

Next, we sought to assess whether transdermally delivered nanosensors could detect disease in this model. Thrombin substrate flanked by near‐IR dye, Cy7, was conjugated to PEG nanoparticles (TDN‐Cy7), were solvated in 30% CAGE and administered topically to Swiss Webster mice. The TDN‐Cy7 nanoparticles were designed such that cleavage of the thrombin substrate would liberate a renally clearable fragment with Cy7 that could enable quantification in the urine by fluorescence. Concomitant quantification of plasma‐borne TDN‐Cy7 was performed to normalize the urine signal in order to mitigate the mouse‐to‐mouse variation that may arise due to the heterogeneous skin conditions present across individual subjects. Thromboplastin was intravenously administered to mice at different time points after topical administration of CAGE/TDN solution on the dorsal skin. Urine collection was performed 30 min after thromboplastin injection, and fluorescence measurement was carried out to detect reporter peptides in the urine. We found that reporter signal was significantly increased (*P* = 0.0079) in the urine of diseased mice as early as 6 h after nanosensor administration (**Figure** **4**A), and that nanosensors retained their diagnostic power at 12 h and 24 hours. When thrombosis was induced at later time points such as 48 hours or 72 hours, the nanosensors could not discriminate diseased from healthy mice. Receiver operating characteristic curves also demonstrated a strong prediction of the thrombosis occurrence at the first 24 h of TDN administration (Figure 4B). In comparison, when TDNs were administered 6 h before the thromboplastin injection, the amount of reporter found in the urine was not significantly different from healthy background, renders the intravenous administration route unsuitable for long term monitoring (Figure 4C,D). These finding indicate that topical administration with CAGE achieves circulatory delivery of nanosensors that enable sustained, noninvasive monitoring of thromboembolism in vivo as opposed to bolus injection into the blood.

**Figure 5:**
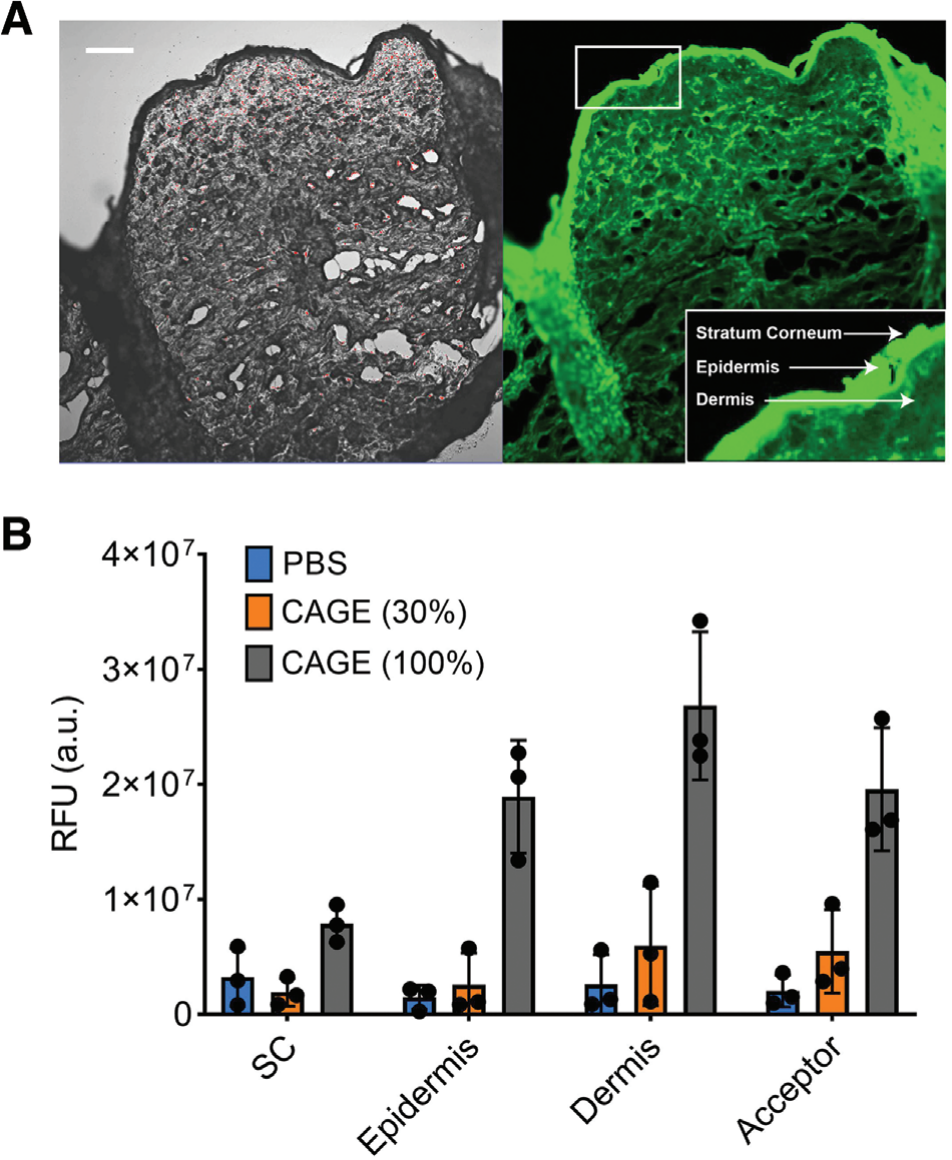
A) Confocal microscopy image of a paraffin‐embedded porcine skin section after 24 h of topical administration of TDN‐qFAM in 100% CAGE. Left: bright field; right: fluorescence image. Scale bar: 200 µm. B) Quantitative results of nanoparticles found in ex vivo porcine skin layers using Franz diffusion cell set‐up with different CAGE concentrations. (mean ± s.d., *n* = 3 independent skin slices).

Finally, as a step toward clinical translation, we sought to assess whether CAGE could facilitate penetration of nanosensors into porcine skin, which is histologically and structurally similar to human skin, with a comparable stratum corneum thickness of 21–26 µm and complete epidermis within the range of 30–140 µm.^[^ ^17^, ^18^ ^]^ Porcine skin samples were placed on Franz diffusion cells, and the diffusion of TDN‐qFAM nanoparticles was tested with different concentrations of CAGE present in the formulation. While a negligible amount of TDN‐qFAM was transported when administered in PBS (Figure S2, Supporting Information), a slight increase in penetration was achieved with 30% CAGE solution (**Figure** **5**). We hypothesized that increasing the CAGE concentration would drive efficient diffusion across porcine skin, which is significantly thicker than mouse skin (epidermis thickness of 10 µm). Accordingly, we found that undiluted (100%) CAGE achieved dramatically improved penetration of TDN‐qFAM through porcine skin. This suggests that our CAGE/TDN coformulation could be modular to attain satisfactory transport efficiency depending on the skin type.

## 3 Conclusion

In this study, we have developed a coformulation of an ionic liquid‐based transdermal delivery agent and thrombosis‐detecting nanosensors to effectively transport a nanodiagnostic agent into the bloodstream. We have demonstrated that the CAGE/nanosensor coformulation is stable and that the cleavage potential of the peptide substrate is not compromised in the presence of CAGE solution. Previous studies indicated that CAGE formulation (when used at 100% concentration) improves the stability and shelf‐life of insulin, likely by coating the outer layer of protein and preventing structural perturbation due to solvation.^[^ ^13^ ^]^ 30% CAGE was used in our experiments to account for the thinner nature and the lower barrier property of mouse skin compared to human and porcine skin. Recombinant thrombin exhibited robust activity against peptide substrates on nanosensors in 30% CAGE formulation, as characterized by in vitro fluorescence measurements.

The local application of CAGE formulation with our TDNs did not initiate any systemic inflammation according to systemic IFN*γ*, IL‐6, MCP‐1, and TNF*α* levels in mouse plasma 1 and 6 h after administration (Figure S3, Supporting Information). This indicates that CAGE percentage in the formulation could easily be adjusted to noninflammatory levels that avoid undesired side effects. Towards clinical applications, however, this percentage should be adapted to human studies because human skin could require higher percentages of CAGE ionic liquids.

We have shown that CAGE enables efficient penetration of TDNs through skin layers both ex vivo and in vivo, enabling sustained, longitudinal monitoring of thrombotic disease. Transdermally administered TDNs could be leveraged to enable at‐home monitoring for thrombosis in hypercoagulable patients following surgery, or rapid detection of thrombotic complications in hospitalized patients, such as those with COVID‐19, who are particularly susceptible to life‐threatening clots.^[^ ^19^ ^]^ Collectively, these results support the clinical development of CAGE:nanosensors for thrombotic disease monitoring, and pave the way for the development of new technologies that leverage topical application to enable non‐invasive diagnosis of the disease.

## 4 Experimental Section

### CAGE Synthesis

Choline geranic acid (1:2) was synthesized as previously reported.^[^ ^13^ ^]^ Briefly, choline bicarbonate (80% in water, Sigma Aldrich) was combined with recrystallized geranic acid (85%, Sigma Aldrich) at 40 °C and left vigorously stirring overnight. The resultant light‐yellow ionic liquid was dried in vacuo at 60 °C at 10 mbar for 2 h, before being placed in a 60 °C vacuum oven for 72 h. The chemical identity of the product was verified with ^1^H NMR Spectroscopy and was in agreement with previously published data.^[^ ^20^, ^21^ ^]^

### Nanosensor Synthesis

Peptides were obtained from Tufts University Peptide Core Facility or CPC Scientific, Inc (USA). For in vitro recombinant thrombin cleavage assays, intramolecularly quenched peptides were used (Table S1, Supporting Information). In vivo thrombin reporter molecules contained urinary reporter Cy7 dye that can be tracked with fluorescence (Excitation 750 nm, emission 790 nm). 8‐arm PEG nanoparticles (40kDa, JenKem Technology) containing maleimide functional groups were dissolved in PBS and filtered through 0.2‐micrometer syringe filters (ThermoFisher). Cysteine terminated peptides (about 20‐fold excess in moles) were added to the PEG solution and allowed to react at room temperature for 8 h. Unreacted peptides were filtered through Amicon centrifugal membranes (30 kDa MWCO, Millipore), and the conjugated nanoparticles were lyophilized for storage. VivoTag750 conjugated PEG nanoparticles were synthesized by mixing *N*‐hydroxysuccinimide functionalized VivoTag750 (20 µL, 10 mg mL^−1^ in DMSO, obtained from PerkinElmer) and 8‐arm PEG‐NH_2_ (5 mg in 0.5 mL PBS) for 12 h at room temperature. Unconjugated VivoTag750 was filtered through Amicon membranes and washed extensively with PBS. After purification, PEG‐nanoparticle content was calculated by a colorimetric assay using ammonium ferrothiocyanate to calculate the ratio of dye:PEG for Cy7 and VivoTag750 molecules.^[^ ^22^ ^]^

### Coformulation Synthesis

The CAGE and TDNs coformulation was prepared by addition of 1 mL of CAGE onto 1 mg of conjugated PEG powder, followed by iterative short mixing and ultrasonication steps until a clear solution was observed. For preparation of additional dilutions, the corresponding amount of PBS was added to reach the desired CAGE:PBS percentage. Because of the tendency of CAGE:PBS mixtures to phase separate, the solution was vortexed briefly to ensure homogeneity prior to application. Size analysis was performed in dynamic light scattering (ZetaSizer), and absorbance spectra were obtained in Tecan Spectrophotometer. For in vitro cleavage assays, recombinant thrombin (10 × 10^−9^ m working concentration) was mixed with TDN‐Q in CAGE (30%) solution (10 × 10^−6^ m by peptide), and cleavage was monitored by fluorescence at 535 nm of dequenched 5‐carboxyfluorescein (5‐FAM). The cleavage study was carried out in mouse serum obtained from Sigma Aldrich).

### Ex Vivo Experiments

The skin penetration studies were undertaken using porcine skin in Franz diffusion cells, as described previously in full.^[^ ^23^ ^]^ Briefly, thawed porcine skin (CBSET, Lexington MA) or mouse skin removed from Swiss Webster strain was placed in a diffusion cell with the stratum corneum facing upwards. The acceptor component of the cell was filled with PBS and equipped with a magnetic stirrer bar. 300 µL was placed on top of the skin, ensuring full coverage. The diffusion cell was placed on a stirrer plate at 37 °C for 24 h, at which point the skin was removed and the surface washed gently with PBS. For quantitative analysis, the stratum corneum was removed by tape stripping (ten layers), the epidermis was separated from the dermis with a scalpel, and a 4 mm punch was used thrice to remove a third of the dermis (by area). The tissue was then placed into a 50% methanol/PBS mixture, and left to shake overnight at RT to extract the drug. 100 µL of the extracted fluid was then placed into a black Corning 96‐well plate and analyzed using a i3 SpectraMax plate reader, with excitation wavelength of 750 nm and an emission wavelength of 775 nm. For the confocal microscopy, the skin was affixed to a cryostat stage with fixing compound and 20 µm slices were taken using a cryostat (Leica CM1950, Leica, Germany). The slices of skin were placed on negatively charged slides (Fisher Scientific, Pittsburgh, PA, USA) and imaged using a confocal microscope (Upright Zeiss LSM 710 NLO) with a 10× air objective.

### In Vivo Experiments

All animal studies were approved by the Massachusetts Institute of Technology (MIT) Committee on Animal Care (protocol 0420‐023‐23). Reporting complied with Animal Research: Reporting In Vivo Experiments (ARRIVE) guidelines. A 1×1 cm^2^ area of dorsal skin of Swiss Webster mice was shaved with an electric razor, and the area was cleaned with sterile PBS‐dampened gauze pads. TDN‐VT (4 nmoles by fluorescent dye) in 10 µL of CAGE (30%) was carefully dispersed on the skin with the help of a rounded tip of a small plastic tube. After 15 min of isolation in a chamber, the excess unsoaked solution was removed with a gauze pad. Blood was collected by retroorbital bleeding using microhematocrit tubes (FisherBrand, 70µL), and then immediately transferred into an equal amount of EDTA (10 × 10^−3^ m in PBS) solution. The plasma was carefully separated from red blood cells after centrifugation at 8000 rcf for 5 min at 4 °C and was stored at −80 °C until further use.

For thrombosis model validation, bovine fibrinogen was reacted with NHS‐VivoTag (Perkin Elmer) for 4 h and purified by Amicon centrifugal membranes (MWCO 100 kDa, Millipore). Labelled fibrinogen and thromboplastin (rabbit, Sigma Aldrich), in varying doses, were intravenously injected with the tail‐vein route to female Swiss Webster mice (4–6 weeks, female, Taconic). After 30 min, mice were euthanized via isoflurane inhalation, and lung, liver, spleen, kidney, and heart were collected. Fluorescence measurements of the extracted organs were performed with LI‐COR Odyssey Infrared Scanner, and the signals were quantified using ImageJ (NIH).

For urine detection of thrombosis, 10 µL of CAGE (30%) containing TDN‐Cy7 nanosensor (4nmoles by peptide) was administered onto previously shaved dorsal skin of Swiss Webster mice, which were subsequently returned to their cages. At different time points postadministration, thromboplastin (0.75 µL g^−1^ bodyweight of 4 mg mL^−1^ stock in PBS) was injected intravenously, and both urine and blood (retroorbital draw) were collected 30 min p.i. Plasma is collected similar to the pharmacokinetic study and both urine and plasma were stored at −80 °C. Cleaved peptide reporters in the urine was quantified with fluorescence of Cy7 (Excitation 750 nm, emission 790 nm). Plasma levels of TDN‐Cy7 were similarly quantified using fluorescence and used for normalizing the urine signals.

For cytokine analysis, 10 µL of CAGE (30%) containing TDN‐Cy7 nanosensor (4 nmoles by peptide) or PBS (as control) were administered onto previously shaved dorsal skin of Swiss Webster mice. Plasma is collected via retroorbital blood draw, after 1 or 6 h postadministration and diluted by 2 times with 10 × 10^−3^ m EDTA PBS solution. The samples are submitted to Eve Technologies, Calgary, Canada for cytokine analysis.

### Statistical Analysis

All samples were prepared in triplicates and each experiment was repeated three times to study variability between experiments. Data are expressed as mean±standard deviation unless differently stated. Results between experimental groups were analyzed by a two‐tailed Mann–Whitney *t*‐test. A *p*‐value < 0.05 was considered significant. Statistical models were made using GraphPad Prism software, version 8.

## Conflict of Interest

S.M. is a shareholder/board member/consultant of Liquideon LLC, CAGE Bio and i2O Therapeutics. S.M. and E.E.L.T. are inventors on patents related to ionic liquids. S.N.B. holds equity in Glympse Bio and Impilo Therapeutics; is a director at Vertex; consults for Cristal, Maverick, and Moderna; and receives sponsored research funds from Johnson & Johnson.

## Supporting information

Supporting Information

## Data Availability

The data that support the findings of this study are available from the corresponding author upon reasonable request.
