# Supplementary material for: Ionic Liquid‐Mediated Transdermal Delivery of Thrombosis‐Detecting Nanosensors
Source: Adv Healthc Mater. 2022 Feb 27;11(11):2102685. doi: 10.1002/adhm.202102685 (PMC11468213; doi:10.1002/adhm.202102685)
Supplement: Supplementary file 1 — Supporting Information [file ADHM-11-2102685-s001.pdf]

ADVANCED HEALTHCARE MATERIALS

Supporting Information

for *Adv. Healthcare Mater.*, DOI 10.1002/adhm.202102685

Ionic Liquid-Mediated Transdermal Delivery of Thrombosis-Detecting Nanosensors

Ahmet Bekdemir, Eden E. L. Tanner, Jesse Kirkpatrick, Ava P. Soleimany, Samir Mitragotri and Sangeeta N. Bhatia**

Supporting Information

Ionic liquid-mediated transdermal delivery of thrombosis-detecting nanosensors

Ahmet Bekdemir[1,2§], Eden E. L. Tanner[3,4§], Jesse Kirkpatrick[1,2], Ava P. Soleimany[1,2,5], Samir Mitragotri[3,6*], Sangeeta N. Bhatia[1,2,6,7,8,9,10*]

1. Koch Institute for Integrative Cancer Research, Massachusetts Institute of Technology, Cambridge, MA, USA
2. Harvard–MIT Division of Health Sciences and Technology, Institute for Medical Engineering and Science, Massachusetts Institute of Technology, Cambridge, MA, USA
1. School of Engineering and Applied Sciences, Harvard University, Cambridge, MA 02138, USA
2. Now at Department of Chemistry and Biochemistry, University of Mississippi, Oxford, MS, 38677, USA
3. Harvard Graduate Program in Biophysics, Harvard University, Boston, MA, USA
4. Wyss Institute of Biologically Inspired Engineering, Harvard University
5. Department of Electrical Engineering and Computer Science, Massachusetts Institute of Technology, Cambridge, MA, USA
6. Department of Medicine, Brigham and Women's Hospital, Harvard Medical School, Boston, MA, USA
7. Broad Institute of Massachusetts Institute of Technology and Harvard, Cambridge, MA, USA
8. Howard Hughes Medical Institute, Cambridge, MA, USA

Email: mitragotri@g.harvard.edu
Email: sbhatia@mit.edu

Nanoparticle	Description
TDN-qFAM	*5FAM*-GGfPRSGGGK*(CPQ2)*-*(EG)$_2$*-C-[8armPEG]
TDN-FAM	*5FAM*-GGfPRSGGGK-*(EG)$_2$*-C-[8armPEG]
TDN-Cy7	*Cy7*-GGfPRSGGGK-*(EG)$_2$*-C-[8armPEG]
TDN-VT	VivoTag750-[8armPEG]

Table S1. Description of nanoparticles used in this work. TDN-qFAM: Quenched thrombosis detecting nanosensors; TDN-FAM: Unquenched 5-carboxyfluorescein conjugated thrombosis detecting nanosensors; TDN-Cy7: Unquenched Cy7 conjugated thrombosis detecting nanosensors; TDN-VT: VivoTag750 labelled PEG nanoparticles; *CPQ2*: Quencher molecule; *5FAM*: 5-carboxyfluorescein. *(EG)$_2$* denotes repetitive ethylene glycol units. Lower case amino acids denote d-stereoisomers.

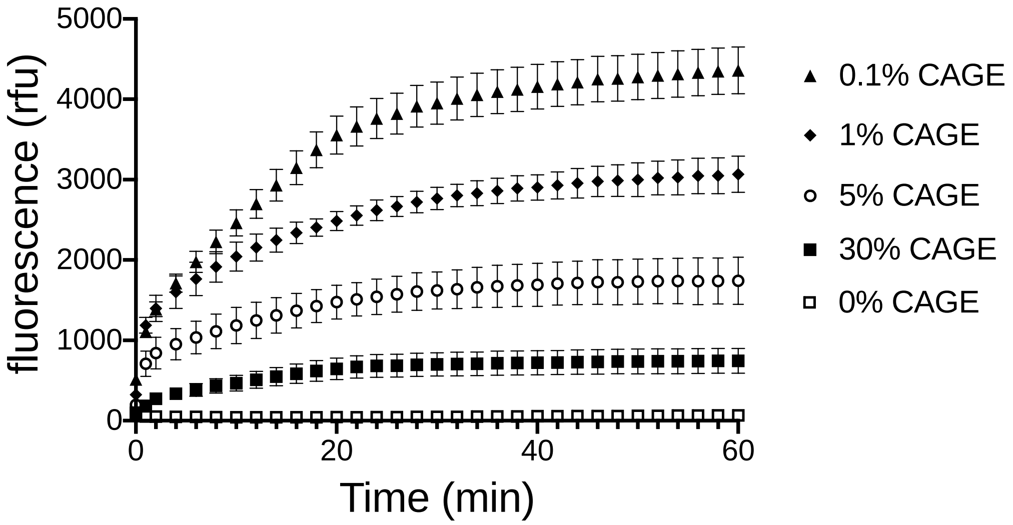

Figure S1. Cleavage kinetics of TDN-qFAM dissolved in different CAGE concentrations and incubated in mouse serum at 37 ºC (mean ± s.d., n =3 independent measurements of the same sample).

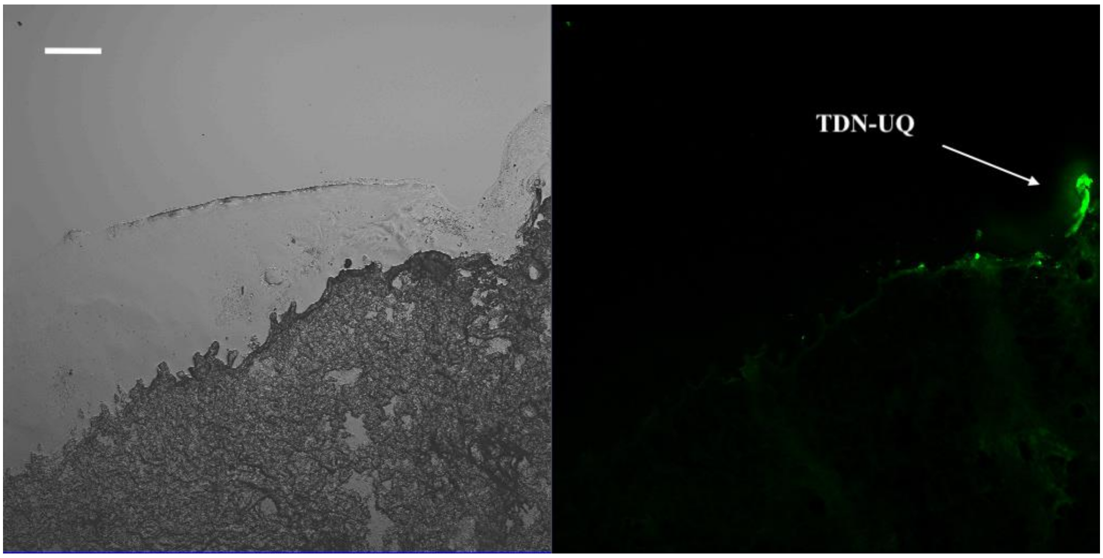

Figure S2. A representative confocal brightfield (left) and fluorescence (right) image of TDN-UQ administered in PBS on porcine skin. The lack of fluorescent signal over the large part of dermis layer indicate that nanosensors in PBS cannot be transported through the skin and mostly stuck at the outermost stratum corneum layer. (Scale bar: 200μm).

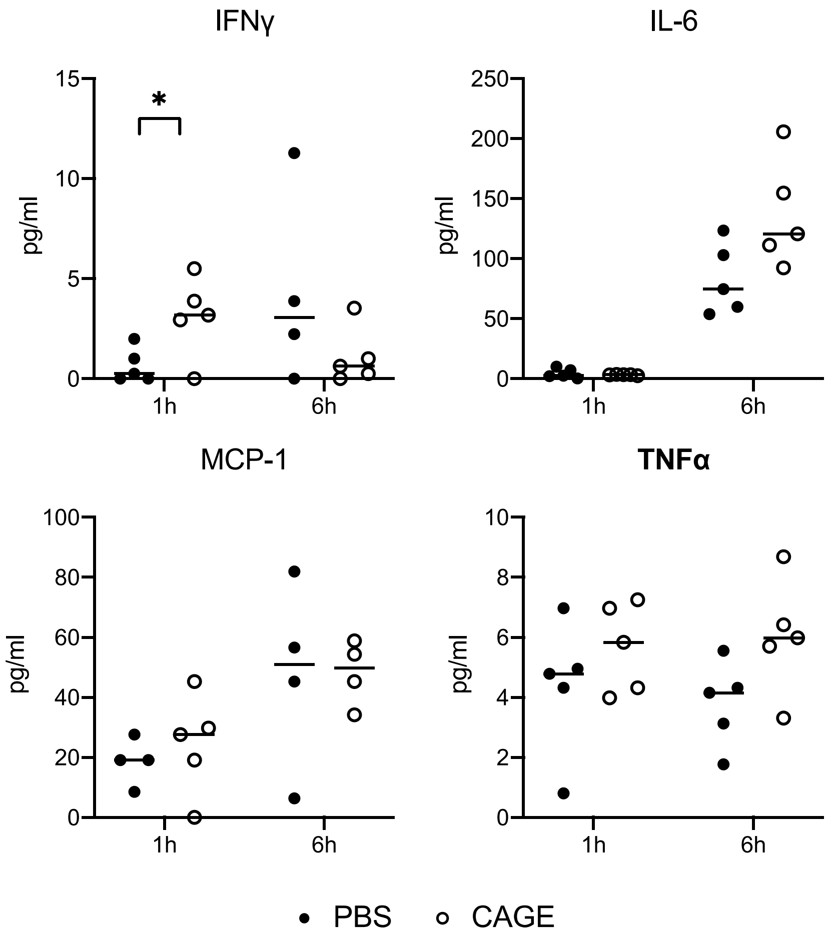

Figure S3. A mouse proinflammatory cytokine analysis for CAGE treated and PBS treated Swiss Webster mice after 1h and 6h of topical administration (lines: mean, n=5 per group). $*P < 0.05$, two-tailed Mann-Whitney's t-test).
